# Supplementary material for: Construction of Charring-Functional Polyheptanazine towards Improvements in Flame Retardants of Polyurethane
Source: Molecules. 2021 Jan 11;26(2):340. doi: 10.3390/molecules26020340 (PMC7826771; doi:10.3390/molecules26020340)
Supplement: Supplementary file 1 [file molecules-26-00340-s001.pdf]

molecules

Supplementary material

Construction of Charring-Functional Polyheptanazine Towards Improvements in Fire safety of Polyurethane

Shaolin Lu, Botao Shen and Xudong Chen *

Key Laboratory for Polymeric Composite and Functional Materials of Ministry of Education, School of Chemistry, Sun Yat-sen University, Guangzhou, 510275, China; lushlin@mail2.sysu.edu.cn (S.L.); shenbt@mail2.sysu.edu.cn (B.S.)

* Correspondence: cescxd@mail.sysu.edu.cn; Tel.: +0086-20-8411-3498

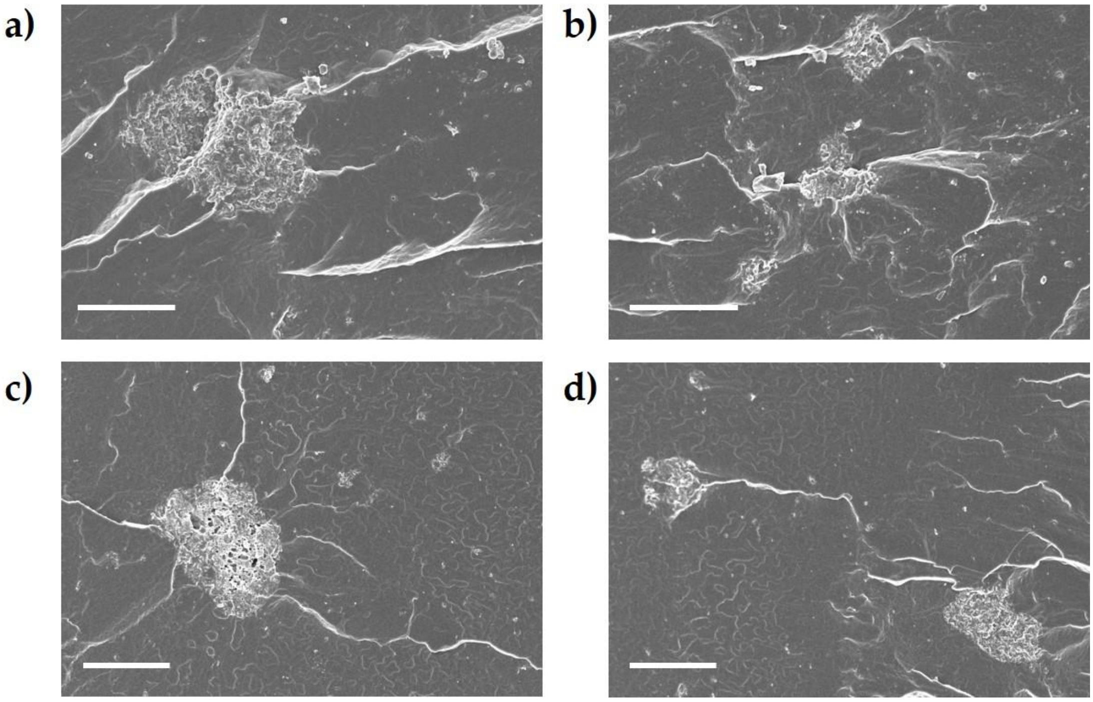

Figure 1. SEM images of fracture surface of (**a**) and (**b**) PCN-TPU under a rotor speed of 60 rpm, (**c**) and (**d**) PCN-TPU under a rotor speed of 100 rpm. Scale bar: 15μm.

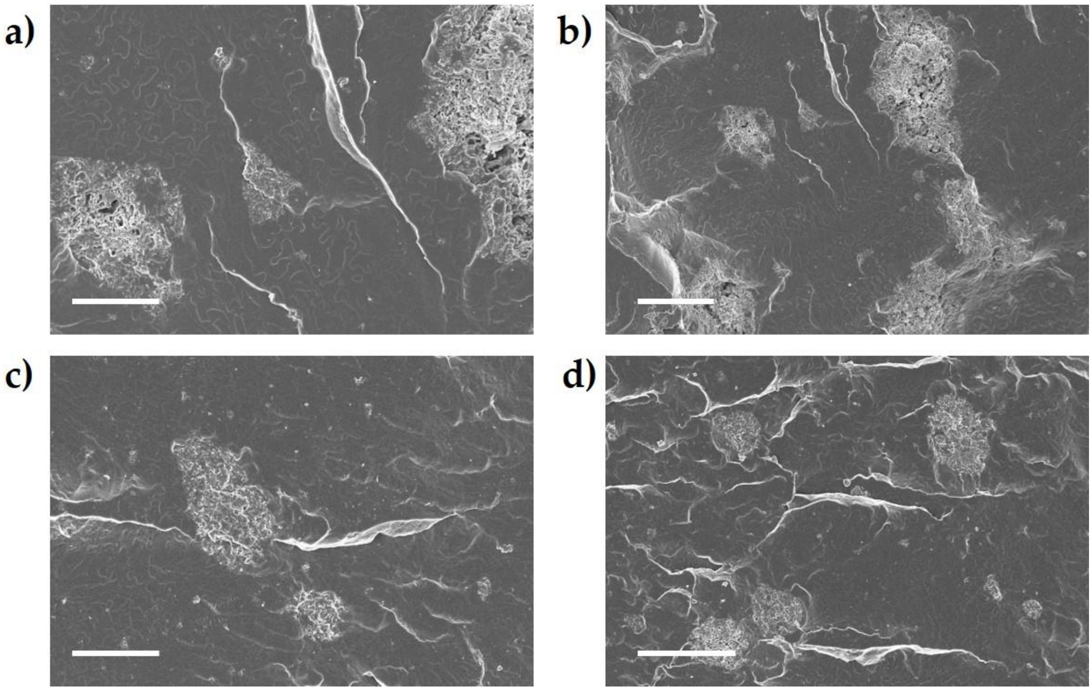

Figure 2. SEM images of fracture surface of (**a**) and (**b**) Co@PCN-TPU under a rotor speed of 60 rpm, (**c**) and (**d**) Co@PCN-TPU under a rotor speed of 100 rpm. Scale bar: (**a**) and (**c**) 15μm, (**b**) and (**d**) 30μm.
